# Exploring HIV Prevention Strategies among Street-Based Female Sex Workers in Chongqing, China

**DOI:** 10.3390/ijerph120100855

**Published:** 2015-01-16

**Authors:** Huan Zeng, Yong Zhao, Siying Meng, Xiaojun Tang, Hang Guo, Yang Wang, Lei Zhang

**Affiliations:** 1 School of Public Health and Management, Chongqing Medical University, Chongqing 400016, China; E-Mails: zhaoyongzy3@sina.com (Y.Z.); tangxiaoj0726@qq.com (X.T.); wangyang8289@163.com (Y.W.); 2 Department of Foreign Language, Chongqing Medical University, Chongqing 400016, China; E-Mail: mpsonia2003@163.com; 3 Institute of Health Policy, College of Human medicine, Michigan State University, Lansing, MI 48910, USA; E-Mail: Guohang@msu.edu; 4 Research Center for Public Health, School of Medicine, Tsinghua University, Beijing 100084, China; 5 The Kirby Institute, University of New South Wales, Sydney, New South Wales 2052, Australia; 6 Melbourne Sexual Health Centre, Alfred Health, Melbourne, Victoria 3053, Australia

**Keywords:** street-based sex worker, Human Immunodeficiency Virus, condom use, commercial sex, peer-based intervention

## Abstract

*Background*: Commercial sex plays an increasingly important role in China’s growing HIV and sexually transmitted infection (STI) epidemics. In China, street-based sex workers (SSWs) are a subgroup of female sex workers with a particularly high risk of HIV/STI infections but are neglected in responses to HIV. This study assesses changes in HIV voluntary counseling and testing (VCT) utilization and high-risk sexual behaviors following a three-month HIV preventive intervention among SSWs in Chongqing, China. *Methods*: A three-month intervention was conducted by a team of peer educators, outreach workers from community-based organizations and health professionals. It mainly included distribution of free pamphlets and condoms and delivery of onsite and clinic-based VCT. Cross-sectional surveys were conducted prior to (*n* = 100) and immediately following (*n* = 112) the intervention to assess its impact. In-depth interviews were conducted among 12 SSWs after the intervention to further explore potential barriers to HIV prevention. *Results:* The intervention significantly increased SSWs’ participation in VCT (from 2.0%–15.2%, *P* < 0.001). Despite participants’ improved HIV-related knowledge level (from 24.0%–73.2%, *P* < 0.001), there were minimal changes in the levels of condom use with clients. Qualitative research revealed that fear of police arrest and stigma were the main barriers to VCT utilization. Low condom use was associated with family financial constraints, inadequate power in condom negotiation, low awareness and misconceptions of HIV infection risks. *Conclusion*: HIV intervention improved VCT utilization and knowledge but we did not observe an increase in condom use after this short intervention. SSWs faced substantial economic, social and environmental barriers to VCT utilization and condom use.

## 1. Introduction

In 2011 alone, 48,000 individuals were diagnosed with Human Immunodeficiency Virus (HIV) in China. An estimated 52.2% of new HIV infections were acquired through heterosexual sex. Condomless sex between female sex workers (FSWs) and their male clients is a major risk of HIV transmission in China [1,2]. Commercial sex work in China has been categorized into various typologies based on where sex is sold (e.g., street-based and venue-based) [3,4]. Establishment-based FSWs work in fixed entertainment venues (e.g., hotels, bars, night clubs, massage parlors and disco houses) and offer sex services in addition to their non-sex work. In contrast, women who solicit clients on streets, in parks and in other public spaces generally rely on sex work as their sole source of income [5]. Street-based sex workers (SSWs) may be more susceptible to HIV infection due to their lower social-economic status [5], frequent high-risk sexual behaviors [6], high levels of sexual transmitted infections (STIs) [7,8] and under-utilization of health care services [9,10].

Although many intervention studies have focused on reducing HIV/STIs risks among establishment-based Chinese FSWs [11,12], few have specifically focused on SSWs. Health outreach, including HIV testing and health education are provided regularly to establishment-based FSWs in many parts of China [12], whereas the needs of SSWs are frequently neglected as they are difficult to reach using conventional campaigns. To address this gap, a three-month pilot intervention was conducted among SSW in Chongqing, China to increase voluntary counseling and testing (VCT) and reduce condomless sex with clients and regular partners. Below we report quantitative results from this intervention study contextualized with qualitative findings on sexual health and risk behaviors from interviews with Chongqing SSWs.

## 2. Methods

### 2.1. Setting and Population

Pre-study field visits and consultation with public health professionals from the Chongqing Centre of Disease Control (CDC) and outreach workers from a local community-based organization (CBO) (“Red Ribbon”), were conducted to generate a map of all known locations where SSW solicited sex. This map was used to select two sites with the highest volume of street-based commercial sex activities within a main urban district (Yuzhong) of Chongqing. Both sites were located near a public square with a high volume of traffic and surrounded by low-end hotels, small grocery shops and restaurants. According to the public health professionals and the outreach workers’ estimation, there were about 120 SSWs in the two sites. We defined SSW as an individual who sold sex on streets, open-areas and public venues (e.g., parks or squares) and had exchanged sex for goods or money within the past 30 days [5]. We excluded sex workers who were establishment-based or did not participate in commercial sex work in the previous month. Two representatives from a local community-based organization (CBO) who had conducted extensive prior outreach work with SSW at these sites, used convenience sampling to recruit participants. Snowballing sampling was also used to recruit additional participants via referral from peer SSWs.

### 2.2. Intervention and Data Collection

A three-month pilot intervention was implemented. The intervention was designed according to the guidelines on HIV prevention and control in China, with specific focus on improving VCT utilization, knowledge, attitudes and safe sex practices about HIV prevention. A pre-intervention workshop was conducted with five health professionals from Chongqing CDC and the STI hospitals of this district, two outreach workers from the partner CBO and four volunteer SSWs. Two experts on HIV prevention were assigned as project supervisors to ensure the quality of the intervention. Intervention was delivered by a team of peer educators, CBO outreach workers and health professionals from CDC and STIs hospitals. It included three parts. In the first part, the four SSWs, who had attended the workshop and were active and good at organizing and educating, were voluntarily selected as peer educators. They were provided with two, two-hour training sessions focused on knowledge and skill-building for HIV prevention and communication. The sessions were delivered by experienced experts and outreach workers from the CDC. A remuneration of $20 was provided to each SSW for their participation in each training workshop. After the sessions, 1000 information booklets with pictorial illustrations of HIV transmission, symptoms, prevention and treatment and 1000 pamphlets, with contact information and the address of the nearest VCT clinics to navigate SSWs’ VCT utilization, were delivered to the peer educators, who were asked to hand out the materials to their fellows. In addition, the peer educators were encouraged to share what they learned from the sessions to their fellows during their daily life and work. In the second part, the CBO outreach workers and SSW peer educators together distributed materials to SSWs in the two selected sites (two public squares), including 8000 high-quality condoms and 6000 booklets and pamphlets. If SSWs were illiterate, the material was verbally explained by the outreach workers. The intervention was conducted twice a month and lasted from 9:00 A.M. to 6 A.M. In the third part, free VCT was provided through two onsite outreach services with mobile vans, each lasted from 9 am to 6pm. Four health professionals from the CDC and STIs hospitals provided the VCT service. Meanwhile, the peer educators and outreach workers helped to mobilize the population. Both the outreach workers and peer educators were asked to keep detailed logs as part of their outreach. According to the work log, the intervention covered 120 SSWs.

The findings of this pilot intervention included quantitative and qualitative components. The quantitative component consisted of two cross-sectional surveys to assess the impact of the intervention. The first was conducted one month before the intervention and the second was delivered at the end of three-month intervention. The survey instrument included 37 questions: 10 on demographic and socio-economic information, 12 on knowledge about and attitudes towards HIV, 8 on sexual behaviors and seven on healthcare-seeking behaviors. In order to investigate some of the structural and social issues underlying quantitative trends [13], after the intervention, in-depth interviews were conducted among 12 SSWs to further investigate the structural and social basis of sexual behaviors. Participants were purposively selected from various educational levels, ages and ethnicities. All of them had been exposed to the intervention. They were recruited by outreach workers or referred by their fellow SSWs. Interviews were conducted in a separate tea house near the sites and each lasted 30–50 min. The qualitative interview included the following domains to explore the social, economic and environmental barriers to SSWs’ VCT utilization and condom use: (1) what do you know about HIV transmission and prevention? (2) What would you do if you are found to be HIV positive and why? (3) What do you know about VCT? If you have not participated in VCT, what is the main barrier? (4) How often do you use condom with your regular partner and why? (5) How often do you use condom with your clients and why? A small gift (~$US 2) was given to each participant after their participation.

### 2.3. Data input and Analysis

Survey data were double-entered using EpiData 3.0 and cross-checked by two investigators. Quantitative data were analyzed using SAS 8.2. Chi-square test or Fisher’s exact test were used to compare the differences in socio-economic characteristics, HIV-related knowledge and the sex behaviors of SSWs, and Mann-Whitney non-parametric test was used to compare the differences between discontinuous variables before and after the intervention.

Qualitative data from interviews were collected and managed according to a standard procedure. Interviews were first audio-taped and later transcribed. The verbatim transcripts were prepared to include interviewee’s expression and manner of speaking. In addition, the interviewer’s own observations about the interview were kept as footnotes at the end of each transcript for later use. Subsequently, investigators read through the transcripts repeatedly and highlighted recurring themes. A thematic framework was established based on themes emerging from the collected data as well as the topic guide used in interviews. All transcriptions were then imported into *MAXqd*a (produced by VERBI GmbH, Berlin Germany) and coded line by line according to the thematic framework. The coded segments relevant to each theme were finally summarized in a chart for data synthesis [14].

### 2.4. Quality Assurance

Both the questionnaire and topic guide for qualitative interviews were developed based on an extensive literature review, previous field work experience and close consultation with experts from Chongqing CDC. Prior to its implementation, the questionnaire was piloted with eight SSWs and three interviews were conducted to confirm the topic guide. Revisions were made accordingly. Both quantitative surveys and interviews were conducted by trained outreach workers and investigators. All the interviews were conducted in private settings to ensure privacy. Quotes used for the qualitative results were firstly translated into English then back-translated into Chinese by two different investigators to ensure consistency.

### 2.5. Ethics Statement

The study protocol was approved by the Institutional Review Board of Chongqing Medical University. All participants provided verbal consent because there was no more than minimal risk associated with participating in this study and no biological samples were collected. All participants signed forms indicating that verbal consent had been obtained for each participant.

## 3. Quantitative Results

### 3.1. Social-Demographic Characteristics

A total of 100 and 112 SSWs participated in pre- and post-intervention surveys, respectively. No significant differences were found across main socio-demographic characteristics when comparing the pre- and post-intervention groups. SSWs aged between 30 and 50 years accounted for 74% (74/100) and 86% (96/112) in the two surveys (Table 1). Before the intervention, the proportion of SSWs who were married was 78% (78/100). Nearly all (90%, 90/100) of the participants had dependent children. More than half (60%, 60/100) had a monthly income less than 2000RMB (~$US 316). Most participants (92%, 92/100) were poorly educated (junior high school or below).

**Table 1:** Demographic and social characteristics of street-based sex workers in Chongqing, Southwest of China, 2008.

Characteristics	Before Intervention *n* (%) *n* = 100	After Intervention *n* (%) *n* = 112	Chi-2 Value ^b^	*P* Value
Age ^a^	--	--	6.92	0.075
<30 years	9 (9.0)	3 (2.7)	--	--
30–39 years	41 (41.0)	45 (40.2)	--	--
40–49 years	33 (33.0)	51 (45.5)	--	--
≥50 years	17 (17.0)	13 (11.6)	--	--
Education	--	--	1.94	0.164
Junior high or below	92 (92.0)	108 (96.4)	--	--
Senior middle or above	8 (8.0)	4 (3.6)	--	--
Current marital status	--	--	5.04	0.162
Not married	10 (10.0)	4 (3.6)	--	--
Married	78 (78.0)	90 (80.4)	--	--
Divorced	8 (8.0)	15 (13.4)	--	--
Widowed	4 (4.0)	3 (2.7)	--	--
Monthly income (RMB)	--	--	2.56	0.278
<2000	60 (60.0)	72 (64.3)	--	--
2000–4000	25 (25.0)	31 (27.7)	--	--
>4000	15 (15.0)	9 (8.0)	--	--
Have children	--	--	2.46	0.117
Yes	90 (90.0)	107 (95.5)	--	--
No	10 (10.0)	5 (4.5)	--	--
Time engaged in sex work	--	--	1.30	0.861
Less than six months	38 (38.0)	44 (39.3)	--	--
Six months-one year	24 (24.0)	21 (18.8)	--	--
One year-two years	21 (21.0)	29 (25.9)	--	--
Two years-three years	8 (8.0)	9 (8.0)	--	--
Three years and above	9 (9.0)	9 (8.0)	--	--
Have ever done sex work off-street	--	--	0.54	0.464
Yes	12 (12.0)	10 (8.9)	--	--
No	88 (88.0)	102 (91.1)	--	--

^a^ Medians of age before and after the intervention were both 40 years old. ^b^ Chi-square test was used to compare social-demographic characteristics before and after the intervention.

### 3.2. Uptake of Voluntary Counseling and Testing

The proportion of participants who knew where and how to access VCT increased from 16.0% (16/100) to 33.9% (38/112), and the proportion reporting participation in HIV VCT services increased from 2.0% (2/100) to 16.1% (18/112) after the intervention. Both changes were statistically significant (Table 2).

### 3.3. Sexual Behaviors with Husbands or Regular Partners

The proportion of participants who did not have sex with their husbands or regular partners in the last month increased from 12.0% (12/100) to 40.2% (45/112) after intervention (*χ^2^* = 29.27, *P* < 0.001). The rates of condom use with husband or regular partner in the last month did not change after the intervention (Table 2), the rates before and after the intervention were 11.4% (10/88) and 13.4% (9/67), respectively.

### 3.4. Sexual Behaviors with Commercial Male Clients

Our analysis showed that the median number of commercial sexual acts in the previous week significantly increased from 10 (IQR 5–20) before the intervention to 15 (IQR 10–23.5) after the intervention (Mann-Whitney test, *χ^2^* = 4175, *P* = 0.005, Table 2). Condom use rates with clients in the last month changed significantly (*χ^2^* = 25.56, *P* < 0.001). Although more SSWs reported using condoms “most of the time” (7.0%, 7/100, *vs.* 33.0%, 37/112, before and after intervention, respectively), the proportion of SSWs who consistently used condoms (means 100% condom use with all clients) decreased from 26.0% (26/100) to 20.5% (23/112). Similarly, the proportion of SSWs who used condoms in their most recent commercial sex act showed no substantial variation after the intervention (*χ^2^* = 1.44, *P* = 0.230). Among those who did not use condoms in their most recent commercial sex, 46.3% (19/41, before intervention) and 51.4% (19/33, after intervention) reported that refusal from male clients was the major cause.

**Table 2:** Uptake of voluntary counseling and testing and sexual behaviors of street-based sex workers before and after intervention, Chongqing, Southwest of China, 2008.

Uptake of Voluntary Counseling and Testing and Sexual Behaviors	Before Intervention *n* (%)	After Intervention *n* (%)	Chi-2 *P*-	
	*n* = 100	*n* = 112	Value	Value
*Uptake of VCT*				
Know where and how to find VCT	16 (16.0)	38 (33.9)	8.95	0.003
Participate in voluntary HIV consultation in the last month	2 (2.0)	18 (16.1)	12.24	0.001
Participate in voluntary HIV test in the last month	2 (2.0)	17 (15.2)	11.25	0.001
*Sexual behaviors*				
Frequency of sex with husband or regular partner in last month	--	--	29.27	<0.001
Once or more per week	63 (63.0)	33 (29.5)	--	--
Less than once per week	25 (25.0)	34 (30.4)	--	--
None	12 (12.0)	45 (40.2)	--	--
Condom use with husband or regular partner in the last month ^a^	--	--	1.50	0.683
Never	40 (45.5)	24 (35.8)	--	--
Sometimes	26 (30.0)	24 (35.8)	--	--
Most of the time	12 (13.6)	10 (14.9)	--	--
Every time	10 (11.4)	9 (13.4)	--	--
Frequency of commercial sex with male clients in the last week	--	--	4175.00	0.005 ^b^
1–5	34 (35.0)	12 (10.7)	--	--
6–10	17 (18.0)	22 (19.6)	--	--
11–15	14 (12.0)	24 (21.4)	--	--
16–20	12 (13.0)	23 (20.5)	--	--
21–25	1 (1.0)	14 (12.5)	--	--
26–30	11 (11.0)	10 (8.9)	--	--
>30	7 (7.0)	7 (6.3)	--	--
missing	4 (4.0)	0 (0.0)	--	--
Condom use with clients in the last month	--	--	25.56	<0.001
Never	13 (13.0)	5 (4.5)	--	--
Sometimes	50 (50.0)	46 (41.1)	--	--
Most of the time	7 (7.0)	37 (33.0)	--	--
Every time	26 (26.0)	23 (20.5)	--	--
missing	4 (4.0)	1 (0.9)	--	--
Condom use in the last commercial sex act	--	--	1.44	0.230
Yes	59 (59.0)	75 (67.0)	--	--
No	41 (41.0)	37 (33.0)	--	--
Reasons of not using condom in the last commercial sex act ^c^	--	--	12.96	0.005
Client refuses to use it	19 (46.3)	19 (51.4)	--	--
SSW regard it as unnecessary	7	1	--	--
Inaccessibility of condom(fail to purchase, too expensive)	3	10	--	--
Others	12	3	--	--
Where do you purchase most of your condoms?	--	--	2.67	0.614
Hospitals	2 (2.0)	2 (1.8)	--	--
Pharmacy	56 (56.0)	65 (58.0)	--	--
Private clinic	3 (3.0)	8 (7.1)	--	--
Sex shops	6 (6.0)	4 (3.6)	--	--
Others	33 (33.0)	33 (29.5)	--	--

^a^ This question was asked among SSW who reported having sex with their husband or regular partner in the last week only, the sampling sizes were 88 and 67 before and after intervention respectively. ^b^ Frequency of commercial sex is not a continuous indicator, the median numbers of sexual acts before and after intervention were 10 (interquatile range: 5–10) and 15 (interquatile range:10–23.5) respectively, Mann-Whitney non-parametric test was used for this indicator instead of Chi-2 test. This p-value corresponds to an analysis based on the original data. ^c^ This question was asked among 41 and 33 participants who indicated not using condom in the last commercial sex act before and after intervention. Fisher's exact test was used to compare reasons in the two surveys. “Others” include forgetting to use condom, having used other contraceptive methods and so on.

### 3.5. Knowledge, Attitude on Prevention of Human Immunodeficiency Virus Infection

The proportion of SSWs who could correctly answer five or more of the seven HIV-related questions (Table 3) tripled from 24.0% (24/100) to 73.2% (82/112) after the intervention (*χ^2^* = 51.18, *P* < 0.001). Prior to the intervention, 39.0% (39/100) of participants indicated that they had no access to HIV-related information (Table 3). Among those who had access, the main sources included friends (31.2%, 19/61), television programs (27.9%, 17/61) and newspaper articles (14.8%, 9/61). In contrast, the inaccessibility rate dropped significantly to 10.7% (12/112, *χ^2^* = 23.13, *P* < 0.001) after the intervention. TV (48.0%, 48/100), free pamphlets (34.0%, 34/100) and peer SSWs (32.0%, 32/100) became the main sources of information following the intervention.

After the intervention, the percentage of participants who would actively seek treatment if diagnosed with HIV infection increased from 78.0% (78/100) to 95.5% (107/112, *χ^2^* = 15.26, *P* = 0.002). The proportion of responders who would choose to “cut off communication” with their friends if their friends become HIV-infected dropped from 75.8%–58.9% (*χ^2^* = 6.75, *P* = 0.034, Table 3).

**Table 3:** Knowledge about Human Immunodeficiency Virus and attitude towards its prevention in street-based sex workers before and after intervention, Chongqing, Southwest of China, 2008.

HIV Knowledge about and Attitude towards HIV/AIDS Prevention and Control	Before Intervention	After Intervention	Chi-2	*P*
	*n* (%) (*n* = 100)	*n* (%) (*n* = 112)	Value	Value
*Knowledge on HIV/AIDS prevention and control*				
Can a person who seems healthy be infected with HIV?	29 (29.0)	67 (59.8)	20.25	<0.001
Can HIV be transmitted by injecting blood or blood products?	46 (46.0)	90 (80.4)	27.12	<0.001
Can HIV be transmitted by sharing needles with people with HIV/AIDS infection?	42 (42.0)	87 (77.7)	28.23	<0.001
Can risks of HIV transmission be reduced by consistent condom use?	47 (47.0)	95 (84.8)	34.17	<0.001
Is it possible for a pregnant woman to transmit HIV to her baby?	43 (43.0)	90 (80.4)	31.54	<0.001
Is it possible to infect HIV by dining with AIDS patients or people with HIV?	22 (22.0)	72(64.3)	38.28	<0.001
Can HIV be transmitted by mosquito bites?	34(34.0)	45(40.2)	0.86	0.353
How do you obtain HIV-related information ^a^	*n* = 61	*n* = 100	69.45	<0.001
Friends	19 (31.2)	21 (21.0)	--	--
Pamphlets	5 (8.2)	34 (34.0)	--	--
TV	17 (27.9)	48 (48.0)	--	--
Fellow SSWs	4 (6.6)	32 (32.0)	--	--
Radio or newspaper	11	13	--	--
Books	8	13	--	--
Billboard	3	15	--	--
Community	5	2	--	--
Doctors or counselling service	7	6	--	--
*Attitude towards HIV infection*				
If you get AIDS, you will	--	--	15.26	0.002
Let it aside	8 (8.0)	3 (2.7)	--	--
Actively participate in treatment	78 (78.0)	107 (95.5)	--	--
Passively participate in treatment	13 (13.0)	2 (1.8)	--	--
Revenge on society	1	--	--	--
If someone around you is infected with HIV, you will ^b^	--	--	6.75	0.034
Cut off communications	75 (75.8)	66 (58.9)		
Treat her/him as usual	15 (15.2)	30 (26.8)		
Care about and help her/him	9 (9.1)	16 (14.3)	--	--
Ever talked about HIV/AIDS with “sisters”	--	--	19.36	<0.001
Yes	28 (28.0)	65 (58.0)	--	--
No	72 (72.0)	47 (42.0)	--	--

^a^ Each participant can have more than one way to obtain HIV-related information. ^b^ One missing data in pre-intervention survey.

## 4. Qualitative Results

Qualitative analysis complemented the quantitative analysis by investigating the determinants of VCT utilization and condom use among the SSW participants. We found that the fear of police arrest and social stigma were the important barriers to VCT utilization and sexual health care. Financial constraints, unwilling male clients, and SSWs’ own misconception about HIV transmission contributed to the low level of condom use among SSWs.

### 4.1. Perception of Fear of Arrest and Stigma

Surprisingly, though five of the 12 SSWs were aware of where and how to access free VCT, none of them really understood what VCT was. Of these 12, 8 were unwilling to attend VCT due to fear of police arrest, despite being informed of the free VCT services. One SSW said:

Interviewer: Do you know ways to obtain free HIV tests?

*Interviewee: What kind of free test? I know nothing about this* (*indicating distress*)*!*

Interviewer: There are free consultations and testing for HIV in CDC clinics, would you like to go?

*Interviewee:* (*Silence*)

*Interviewer: How about your “sisters”* (*referring to fellow SSWs*)*?*

*Interviewee: Possibly they will not go. They are afraid of being arrested by policemen* (*Silence again*).

(SSW-7, 45 years old)

Perceived stigma and discrimination from health care providers also imposed substantial barriers to SSWs’ timely utilization of sexual health care.

*I know some sisters and Bangbang* (local dialect for “male casual labourers”)*, they suffered some serious sexual transmitted infections. It was so serious that they had to go back to their rural hometown* [*for treatment*]. *Because they felt ashamed to visit doctors here they are afraid that they may be looked down upon by others.*

(SSW-4, 40 years old)

### 4.2. Financial Constraints

Financial constraints resulting from SSWs’ heavy family burdens and low incomes were prominent themes among SSWs explaining the initiation and continuation of participating in sex work. Interviews indicated that SSWs had started sex work as a result of an extended period of unemployment due to illiteracy and the heavy financial burdens due to expenses of children’s living and education.

*For people like us, we just have limited education* (*regretfully*). *So it’s hard for us to earn a living. We have no other choices.*

(SSW-7, 45 years old)

Besides, I have two children at school…In fact, who would like to do this! That’s all because we have no other choices. My husband is a peasant; he can’t make a living. As for me, since I left my hometown, for a long period of time, I couldn’t find a job, so I have to do this “business”.

(SSW-2, 38 years old)

Although some participants became aware of the risks of HIV infection as a result of the intervention, they still chose not to use condoms in order to maintain their business.

*In the past, I was very afraid of AIDS. How terrible if you infect HIV! But now I am not so afraid about it. Because they* (*referred to the CBO outreach workers and the SSW peers*) *told us that talking, eating and handshaking could not transmit HIV. There are only three ways of infection, as sex, from mother to child and blood transmission.*

(SSW-12, 48 years old)

*The clients really do not want to use condom, what can I do?* (*Indicating upset*) *I have to meet their needs.*

(SSW-2, 38 years old)

If you insist on using condom, they will just say goodbye.

(SSW-3, 40 years old)

Additionally, SSW interviewees indicated that low awareness or negative attitude towards HIV prevention among clients, especially those who were older or from rural areas, often led to refusing condom use.

*However, those from the countryside have no idea about it* (*condom use*), *so generally, they refuse to use condom.*

(SSW-1, 37 years old)

*In fact, it is the clients who refuse* (*using*) *condoms; they say it is not comfortable and decreasing their pleasure. We really want them to use it. After all, who would like to get the diseases? …In general, younger clients are more likely and willingly to use condom, but the old men will not, because they think they are old enough, they are not afraid of death given the incubation period* (*of AIDS*) *is very long.*

(SSW-6, 42 years old)

SSW’s low income also limited their ability to purchase quality condoms and imposed a barrier for condom use. As one respondent described:

*I do not have so much business* (*recently*), *so I do not earn enough. That is why I only buy the cheapest condoms, at the price of* (*a pack of*) *20 condoms for one RMB. But these cheapest ones break easily. However, I cannot afford the more expensive ones. Sometimes I can only earn ten RMB from one transaction because I am looking old, but the younger ones who look pretty could earn more, they could earn 50 to 100 RMB for one transaction.*

(SSW-7, 45 years old)

### 4.3. Low Awareness and Misconceptions on Risks of Human Immunodeficiency Virus Infection

Four interviewees also demonstrated misconceptions about HIV transmission from male clients. SSWs often perceived themselves as “safe” from HIV infection because their clients were mainly locals.

*I do not think we will get AIDS, because we are not drug users. So far, I have not heard about AIDS in Chongqing; this is* (*something*) *more in foreign countries. The sex workers in hotels can easily acquire HIV, because their clients’* (*social background*) *are complicated, coming from all different countries and areas. But our clients are sampled nearby, including the old, the retired and Bangbang. We will not get AIDS, we are safe.*

(SSW-8, 45 years old)

Further misconception, that SSWs perceived HIV infection might be preventable by using disinfectants and anti-inflammatory drugs, still existed even after intervention.

*I think the disinfectants can prevent the disease* (*HIV*); *I think that is enough. Besides, we often take anti-inflammatory drugs.*

(SSW-2, 38 years old)

## 5. Discussion

This study describes an intervention targeting a highly marginalized subgroup of SSWs who are vulnerable to HIV/STIs. Although interventions have focused on HIV prevention among FSWs in China [12,15,16], to our knowledge this is the first report of an intervention study specifically targeting SSWs. The international literature has indicated that homelessness, high risk behavior, low health knowledge and accessibility to healthcare are major health issues for SSW [5,7,9], but the unique needs of the SSW population demand tailored responses in order to overcome the complex social and cultural challenges. Our intervention is unique in the Chinese context as it acknowledged the low social status of SSWs, explicitly involved sex workers during the intervention and received direct support from local NGOs and CDC.

Our study reveals a remarkably low utilization of VCT among SSWs. The 2.0% utilization rate prior to intervention is similar to the level in the general Chinese population (2.3%–6.0%, [17,18]). Although the post-intervention utilization rate has significantly increased (15.2%), it remains substantially lower than that among the general FSW population in both China (27%–48% [5,19,20,21]) and other international settings (~90% in Thailand and Guinea [22,23]). Notably, although over one-third of the study participants know how to access VCT after the intervention, only half of them actually obtain it. The fear of police arrest and stigma from health providers are the major barriers, and concerns about privacy, mistrust of the program staff and fear of possible positive test results are also significant obstacles for accessing VCT [24,25,26].

Our health education intervention improved knowledge and attitudes about HIV infection, and increased self-reported HIV testing, but did not decrease condomless sex. In comparison, previous similar intervention studies among a general FSW population in China have reported both a significant improvement of HIV knowledge and sexual behavioral changes with commercial clients [1,15]. Our qualitative interviews reveal that clients’ unwillingness to use condoms, SSWs’ heavy family financial burdens, insufficient power to negotiate condom use, low awareness and misconceptions about HIV infection are the underlying causes. Family financial difficulties may be the key driver among the various factors. This is evidenced by the demographic characteristics of our participants who consistently exhibit lower income and a higher proportion (>90%) of dependent children compared with general FSWs [5]. Therefore, SSWs may face more formidable obstacles than general FSWs for behavior changes during intervention in China; this is consistent with studies in other countries [7,8].

Our analysis shows that the median number of commercial sexual acts in the previous week significantly increased from 10 before the intervention to 15 after the intervention. This is consistent with the median range (6–14 acts in the last week) of other SSW studies in China [5,27,28]. The lower acts in the baseline study may relate to the crackdown on sex work before the baseline survey. Interestingly, this study found that the proportion of participants who did not have sex with their husbands or regular partners in the last month increased from 12.0% before intervention to 40.2% after intervention. One possible reason was that after the intervention, SSWs understood the risks of not using condoms during sex, but their husbands or regular partners often did not like use condoms, so SSWs choose less sex with them. Also, when SSWs had more sex with clients, their sex with husbands or partners would decrease.

A number of limitations of this study should be noted. Firstly, the sample size of the recruited participants is small due to the marginalized nature and limited accessibility to SSWs in China. Secondly, SSWs who participated in this study are likely to represent an isolated subgroup keen to receive support and HIV prevention; as a result, they are more likely to be selected for this study by our convenient sampling method. Besides, interviewers were also more likely to select the same participants at post-intervention survey again if they have already established links in the first survey. Thirdly, when being asked about their risk behaviour, study participants may be prone to provide socially desirable answers to the interviewers, leading to potential biases in the findings. Fourthly, this study lacks an adequate control group to accurately evaluate the intervention, and the absence of practice effect correction may bias the results of intervention. Finally, for privacy protection consideration, this study did not collect personal identity identifiers to enable one-to-one matching and a paired test before and after the intervention. However, according to the outreach workers, the SSWs in the two sites were relatively stable in the three months and the intervention reached most of them. Since the possible newcomers were only partly engaged in the intervention, if at all, and the SSWs who left our study had received the intervention, the potential mobility in our study participants may have caused us to under-estimate the actual impacts of intervention.

## 6. Conclusions

This study demonstrates that Chinese SSWs in Yuzhong District, Chongqing have a low utilization of VCT and face substantial economic, social and environmental barriers to behavioral changes and HIV prevention; the unique characteristics and needs of this population require tailored responses in order to overcome these complex challenges. While SSW peer leader-based interventions combined with efforts from CBO and CDC may be one effective approach for increasing HIV testing and HIV knowledge among some SSWs, more complex interventions that address financial constraints, lack of empowerment and condom negotiation skills, and education of SSW clients are warranted. Multi-level approaches that address these entrenched factors are essential for effective HIV preventions among this hard-to-reach population in China [29,30].
